# The factor inhibiting HIF regulates T cell differentiation and anti-tumour efficacy

**DOI:** 10.3389/fimmu.2024.1293723

**Published:** 2024-04-16

**Authors:** David Bargiela, Pedro P. Cunha, Pedro Veliça, Lena C. M. Krause, Madara Brice, Laura Barbieri, Milos Gojkovic, Iosifina P. Foskolou, Helene Rundqvist, Randall S. Johnson

**Affiliations:** ^1^ Department of Physiology, Development and Neuroscience, University of Cambridge, Cambridge, United Kingdom; ^2^ Department of Cell and Molecular Biology, Karolinska Institutet, Stockholm, Sweden; ^3^ Department of Laboratory Medicine, Karolinska Institutet, Stockholm, Sweden

**Keywords:** hypoxia-inducible factor, factor inhibiting HIF, T cells, immunotherapy, imunometabolism

## Abstract

T cells must adapt to variations in tissue microenvironments; these adaptations include the degree of oxygen availability. The hypoxia-inducible factor (HIF) transcription factors control much of this adaptation, and thus regulate many aspects of T cell activation and function. The HIFs are in turn regulated by oxygen-dependent hydroxylases: both the prolyl hydroxylases (PHDs) which interact with the VHL tumour suppressor and control HIF turnover, and the asparaginyl hydroxylase known as the Factor inhibiting HIF (FIH), which modulates HIF transcriptional activity. To determine the role of this latter factor in T cell function, we generated T cell-specific FIH knockout mice. We found that FIH regulates T cell fate and function in a HIF-dependent manner and show that the effects of FIH activity occur predominantly at physiological oxygen concentrations. T cell-specific loss of FIH boosts T cell cytotoxicity, augments T cell expansion *in vivo*, and improves anti-tumour immunotherapy in mice. Specifically inhibiting FIH in T cells may therefore represent a promising strategy for cancer immunotherapy.

## Introduction

Oxygen has a central role in driving metabolic and cell fate-determining programmes. Within cells, the HIF transcription factor orchestrates an adaptive transcriptional response to maintain oxygen homeostasis by altering its availability and utilisation within cells. Control of this process is achieved through two disparate sets of oxygen-sensing hydroxylase enzymes: the first, the prolyl hydroxylases (PHD), which interact with the VHL tumour suppressor complex and regulate proteasomal turnover of HIF; and the second, the significantly less studied Factor inhibiting HIF (FIH). The FIH asparagine hydroxylase regulates the transcriptional activity of HIF through controlling HIF association with the transcription co-activator CBP/P300 (1). PHD inhibitors that modulate systemic HIF levels have demonstrated clinical efficacy in a number of settings, including the treatment of anaemia in patients with chronic kidney disease (2). We have recently shown that one member of this class of compounds can increase T cell cytotoxicity and anti-tumour function *in vivo* (3). Although less well understood, it is clear that FIH can as well modulate HIF signalling across a wide range of oxygen tensions (4, 5), including those encountered in hypoxic tumour or inflammatory environments (6).

Our group and others have demonstrated that modulation of HIF activity in CD8+ T cells can improve anti-tumour immune responses (7–13). HIF-1α depletion does not impact T cell maturation (14, 15) but significantly hampers TCR activation and antitumour T cell function (11, 16). Consistent with this, T cells in which VHL or all three PHDs (PHD1-3) are knocked out have increased HIF levels, boosted effector responses and confer improved anti-tumour control.

The role of FIH in T cell responses has not previously been explored. Previous studies in mice, using both global and tissue-specific FIH knockouts, indicate that FIH loss results in a distinct metabolic profile, altering oxygen utilisation and cell fate in a HIF-dependent manner (17, 18). In mice with skeletal muscle lacking FIH, oxygen consumption increases during exercise under normal conditions but decreases in low oxygen environments (12% oxygen) (18). While at the cellular level, FIH-null fibroblasts exhibit higher basal and maximum cellular respiration (18). In T cells, its relevance to modulating HIF activity and cell fate was suggested by a recent overexpression study that showed loss of FIH regulation of HIF acts to increase T cell cytotoxic efficacy (9). Here, we assess the role of FIH directly in T cells using a genetic model of loss of function, via a T cell-specific knockout of FIH. We demonstrate that targeting FIH activity alters both T cell function and fate, and that FIH deletion in T cells improves anti-tumour immunotherapy.

## Results

### FIH regulates HIF activity in T cells

**Figure 1:**
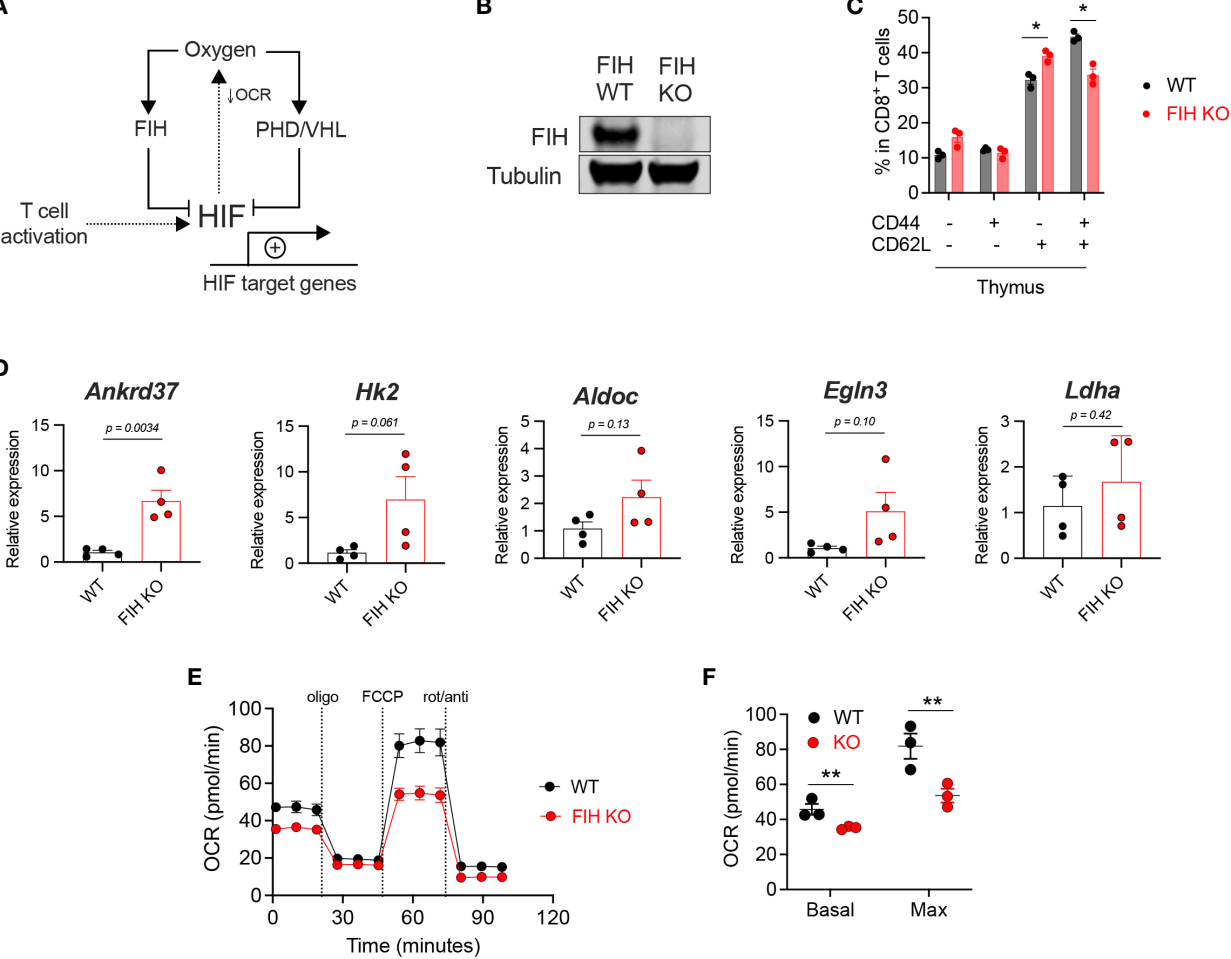
Loss of FIH increases HIF activity in T cells. **(A)** HIF target gene expression is regulated by oxygen levels and signals downstream of the T cell receptor following activation. Oxygen-dependent enzymes regulate the amount (PHD, with VHL) and the transcriptional activity (FIH) of HIF. HIF activity reduces oxygen consumption rate (OCR) by limiting mitochondrial oxidative phosphorylation. **(B)** Immunoblot of FIH protein expression in *Hif1an* ^fl/fl^ *dLck* Cre negative (‘WT’) and *Hif1an^f^* ^l/fl^ *dLck* Cre positive (‘FIH KO’) CD8+ T cells. **(C)** Frequency of CD44-CD62L-, CD44+CD62L-, CD44-CD62L+ and CD44+CD62L+ cells as a proportion of total thymus CD8+ T cells in WT (n=3) and FIH KO (n=3) mice. **(D)** Expression of known HIF target genes in day 3 activated WT and FIH KO CD8+ T cells (n=3 mice per genotype) measured by qRT-PCR. **(E)** Seahorse metabolic profile showing oxygen consumption rate of activated WT and FIH KO CD8+ T cells (n=3 mice per genotype). **(F)** Basal and maximal oxygen consumption rates in activated WT and FIH KO CD8+ T cells (n=4 mice per genotype). Error bars denote s.e.m. Multiple unpaired t tests with Šídák’s multiple comparison test **(C)**. Unpaired t test **(D)**. Two-way ANOVA with Šídák’s multiple comparisons test **(F)**. *p<0.5, **p<0.01. FIH, factor inhibiting HIF; OCR, oxygen consumption rate; PHD, prolyl hydroxylases, VHL, von Hippel-Lindau protein.

HIF-driven gene expression increases almost immediately following T cell activation. Prolyl hydroxylases (PHDs), together with the ubiquitin ligase von Hippel Lindau (VHL), regulate HIF abundance, while factor-inhibiting HIF (FIH) regulates HIF transcriptional activity (1). As can be seen, HIF-driven metabolic responses, including the suppression of mitochondrial oxidative phosphorylation, reduce mitochondrial oxygen consumption and maintain redox homeostasis (**Figure 1A**) (19, 20).

To address the role of FIH function in T cells, we generated a T cell-specific (distal *Lck* (*dLck*) Cre) FIH knockout mouse model (**Supplementary Figure 1A**). We confirmed gene deletion by measuring FIH protein levels in CD8+ T cells obtained from *Hif1an* ^fl/fl^ *dLck* Cre negative (FIH wild-type, ‘WT’) and *Hif1an^f^* ^l/fl^ *dLck* Cre positive (FIH knockout, ‘FIH KO’) mice (**Figure 1B**). As shown here, loss of the FIH gene in this model results in an essentially complete removal of FIH protein expression in isolated primary T cells. Using publicly available datasets, we found increased FIH protein expression in activated mouse CD8+ T cells (**Supplementary Figure 1C**), without significant changes in Hif1an gene expression (**Supplementary Figure 1B**).

To assess the effect of T cell-specific FIH loss *in vivo*, we profiled T cell populations in secondary lymphoid organs, including the thymus, lymph node and spleen, in WT and FIH KO mice. In the thymus, loss of FIH in T cells resulted in a modest reduction in CD8+ central memory (CD44+CD62L+) cells and an increase in CD8+ naïve (CD44-CD62L+) cells (**Figure 1C**). There were no significant differences in other T cell populations (**Supplementary Figures 1B–E**) in these sites.

To determine the role of FIH in regulating HIF transcriptional activity in T cells, we measured mRNA expression of known HIF target genes using qRT-PCR in WT and FIH KO CD8+ T cells. We found that the expression of HIF target genes was not significantly different in WT and FIH KO CD8+ T cells activated in 21% oxygen, apart from *Ankrd37*, which had significantly higher expression in FIH KO T cells (*p*<0.01) (**Figure 1D**).

A key metabolic outcome of HIF-driven gene expression is to limit cellular consumption of oxygen, particularly by restricting mitochondrial oxidative phosphorylation (21). We measured oxygen consumption rates (OCR) and extracellular acidification rates (ECAR) and found that activated FIH KO CD8+ T cells had significantly lower rates of basal and maximal oxygen consumption when compared to WT CD8+ T cells (**Figures 1E, F**) at atmospheric oxygen conditions. No significant differences in ECAR were noted between WT and FIH KO CD8+ T cells also at atmospheric oxygen conditions (**Supplementary Figures 1F, G**).

### FIH alters T cell function and fate in a HIF-dependent manner

**Figure 2:**
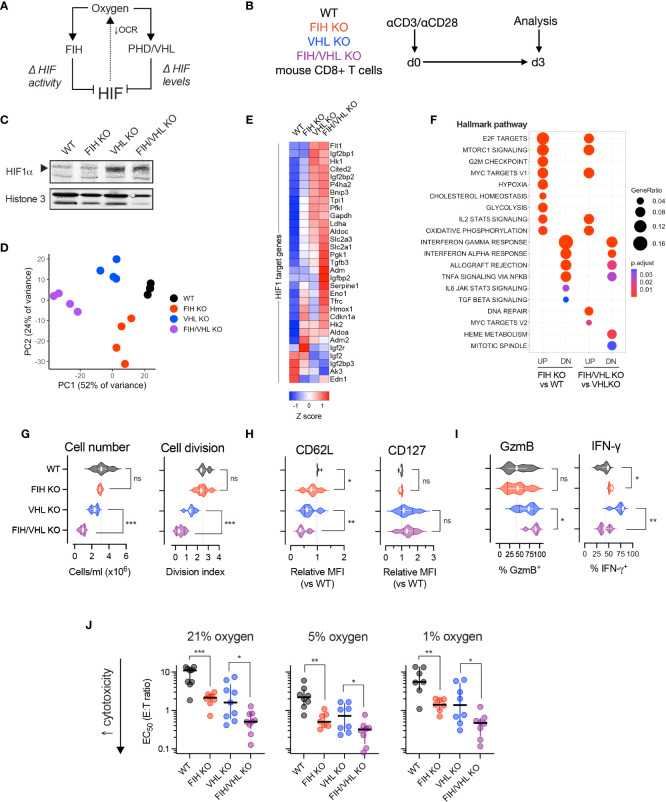
FIH-driven modulation of T cell function is oxygen- and HIF-dependent. **(A)** HIF is regulated by the oxygen-dependent hydroxylases, FIH and PHD. FIH regulates HIF transcriptional activity and PHDs, together with VHL, regulate HIF amount. **(B)** Experimental design – WT, FIH KO, VHL KO and FIH/VHL double KO mouse CD8+ T cells were activated using anti-CD3/CD28 microbeads then cultured at 21% oxygen (unless otherwise specified) for 3 days prior to being analyzed. **(C)** Immunoblot showing HIF-1alpha (HIF-1α) protein levels in WT, FIH KO, VHL KO and FIH/VHL CD8+ T cells. **(D)** Principal components analysis (PCA) of RNAseq expression data from WT, FIH KO, VHL KO and FIH/VHL CD8+ T cells (n=4 mice per genotype). **(E)** Z score gene expression of HIF1 target genes (from Semenza HIF1 target gene set (22)) in WT, FIH KO, VHL KO or FIH/VHL CD8+ T cells (n=4 mice per genotype). **(F)** Pathway enrichment analysis using mSigDB Hallmark dataset to evaluate genes significantly upregulated (UP) and downregulated (DN) in FIH KO vs WT and FIH/VHL KO vs VHL KO comparisons. **(G–I)** Proliferation assessed by cell number and cell division **(G)**, memory markers **(H)** and effector molecules **(I)** in WT, FIH KO, VHL KO and FIH/VHL CD8+ T cells. **(J)** EC50 values from *in vitro* cytotoxicity assay of WT, FIH KO, VHL KO and FIH/VHL CD8+ T cells at 21%, 5% and 1% oxygen. Error bars denote median with interquartile range. Unpaired t test **(G–J)**. *p<0.05, **p<0.01, ***p<0.001; ns, non-significant.

HIF-driven gene expression depends on HIF abundance (regulated by PHD/VHL) and HIF transcriptional activity (regulated by FIH). To determine the role of FIH in T cell fate and function we assessed proliferation, differentiation and *in vitro* cytotoxicity of CD8+ T cells while varying oxygen and HIF levels, using a combination of FIH and VHL gene deletions (**Figures 2A, B**).

We first measured HIF-1alpha (HIF1α) protein levels in nuclear extracts from day 3 activated WT (*Hif1an* ^fl/fl^ *dLck Cre-*), FIH KO (*Hif1an* ^fl/fl^ *dLck Cre+*), VHL KO (*Vhl* ^fl/fl^ *dLck Cre+*) and FIH/VHL KO (*Hif1an* ^fl/fl^ *Vhl* ^fl/fl^ *dLck Cre+*) CD8+ T cells. As expected, we found low HIF1α protein levels in WT and FIH KO cells and high HIF1α protein levels in VHL KO and FIH/VHL KO cells (**Figure 2C**). To determine how this translated to changes in HIF-driven gene expression we performed RNAseq on WT, FIH KO, VHL KO and FIH/VHL KO CD8+ T cells. Principal components analysis (PCA) of gene expression levels separated samples according to genotype (**Figure 2D**); and using *k*-means clustering, we were able to identify discrete gene clusters (**Supplementary Figure 2A**) and pathway enrichment profiles (**Supplementary Figure 2B**) that varied according to genotype. We compared the expression of HIF1 target genes across each genotype and found that the majority of genes showed a step-wise increase in expression, with the lowest expression in WT CD8+ T cells and the highest expression in FIH/VHL KO CD8+ T cells (**Figure 2E**). Some genes showed the opposite pattern, with highest expression in WT cells and lowest expression in FIH/VHL KO, while others were highly expressed only in the context of FIH deletion (in FIH KO or FIH/VHL KO CD8+ T cells) (**Figure 2E**).

We performed differential expression analysis to identify significantly upregulated/downregulated genes (adjusted p value <0.05) between the differing gene deletions and wild-type CD8+ T cells. Over half of all genes that were significantly upregulated (2914/5550, 52.5%) or downregulated (2838/4997, 56.8%) were shared between knockout genotypes (**Supplementary Figure 2C**). For the purposes of this study, we focussed on genes whose expression changed significantly following FIH loss at either low or high HIF levels (FIH KO versus WT or FIH/VHL KO vs VHL KO, respectively). We found specific pathways that were enriched following FIH loss at low HIF levels only, or at high HIF levels only (**Figure 2F**). We also noted pathways that were enriched following FIH loss at both low and high HIF level (**Figure 2F**).

We next profiled the effects of FIH activity on T cell proliferation and differentiation. In VHL-wildtype T cells, where HIF levels are lower, FIH loss resulted in no changes to cell proliferation (**Figure 2G**), but gave rise to reduced CD62L expression, albeit with no changes to CD127 (**Figure 2H**). FIH loss increased expression of interferon-gamma (IFNγ), but not Granzyme B (GzmB) (**Figure 2I**). Whereas, when VHL was absent (VHL KO and FIH/VHL KO) and HIF levels were higher, FIH loss resulted in a further reduction in cell proliferation; loss of the memory marker CD62L but not CD127; and higher expression of Granzyme B (GzmB) but lower expression of interferon-gamma (IFNγ) (**Figures 2G–I**).

We compared *in vitro* cytotoxicity of T cells of each genotype at high (21%), intermediate (5%), and low (1%) oxygen tensions. We found that FIH loss across all oxygen tensions and at both low and high HIF levels (when VHL is present or absent, respectively) significantly increased *in vitro* cytotoxicity, with maximal effects noted at intermediate (5%) oxygen levels (**Figure 2J**, **Supplementary Figures 2A–C**).

Overall, loss of FIH alters T cell function and fate in a HIF-dependent manner. At low HIF levels (VHL present), loss of FIH in T cells boosts effector differentiation and interferon gamma expression. At high HIF levels (VHL absent), loss of FIH further boosts effector differentiation, but results in reduced *in vitro* proliferation and reduces interferon gamma expression. We found that *in vitro* cytotoxicity is boosted by FIH loss across a range of oxygen and HIF levels.

### FIH activity is maximal at intermediate oxygen levels and indirectly regulates other oxygen-dependent enzymes

**Figure 3:**
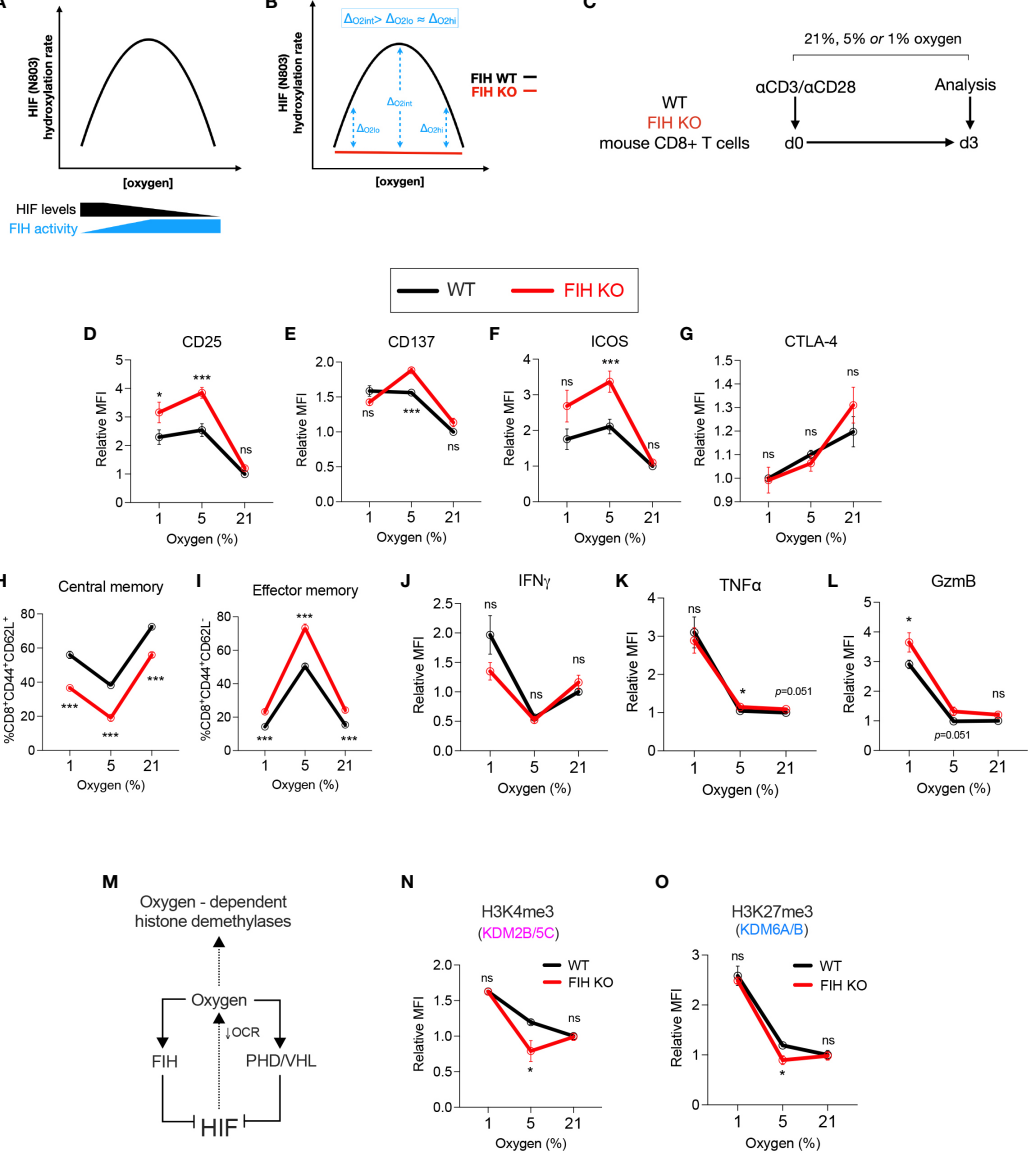
FIH activity is maximal at intermediate oxygen levels. **(A)** Graph illustrating predicted amounts of FIH-mediated hydroxylation of HIF across a range of FIH substrate levels (HIF and oxygen). **(B)** Graph illustrating predicted maximal differences in FIH activity at intermediate oxygen (O_int_) when compared to low (O_lo_) or high (O_hi_) oxygen concentration. **(C)** Experimental design – WT and FIH KO mouse CD8+ T cells were activated using anti-CD3/anti-CD28 microbeads then cultured at 21%, 5% or 1% oxygen for 3 days prior to being analysed. **(D–K)** Activation/checkpoint markers **(D–F)**, differentiation markers **(G, H)** and effector molecules **(I–K)** on WT and FIH KO mouse CD8+ T cells measured using flow cytometry. **(L)** The intracellular oxygen pool is shared by both oxygen-dependent histone demethylases and oxygen-dependent HIF hydroxylases (FIH, PHD) and regulated by HIF activity via changes in mitochondrial oxygen consumption rate (OCR). **(M, N)** Expression levels of H3K4me3 **(M)** and H3K27me3 **(N)** (demethylated by Kdm2b/5c and Kdm6a/b, respectively) on WT and FIH KO mouse CD8+ T cells measured using flow cytometry. Error bars denote mean with s.e.m. Two-way ANOVA with Šídák’s multiple comparisons test **(D–K, M, N)**. *p<0.05, ***p<0.001; ns, non-significant.

We next evaluated whether FIH activity varies depending on substrate/co-substrate availability. Assuming stable expression of FIH, FIH-mediated regulation of HIF transcriptional activity would be predicted to vary according to two inter-related factors – substrate availability (i.e., HIF levels, regulated by PHD/VHL) and co-substrate availability (oxygen, alpha-ketoglutarate, Fe2+). FIH has been shown to have a higher affinity for oxygen when compared to the PHD enzymes (23, 24), and continues to hydroxylate and inhibit HIF activity at lower oxygen concentrations, including those where PHD activity is absent, and thus HIF protein levels are maximal (**Figure 3A**). Therefore, when comparing WT to FIH KO T cells, the largest effect size from FIH activity would be predicted to occur at intermediate oxygen tensions, where the combination of substrate (HIF levels) and co-substrate (oxygen) availability is maximised (**Figure 3B**). To test this hypothesis, we cultured WT and FIH KO CD8+ T cells in high (21%), intermediate (5%) and low (1%) oxygen tensions for 3 days and measured T cell differentiation and epigenetic markers (**Figure 3C**).

In keeping with our hypothesis, we found that markers of T cell activation – CD25, CD137 and ICOS - were significantly increased in FIH KO T cells when compared to WT cells when cultured at 5% oxygen, but not at 21% or 1% oxygen (**Figures 3D–F**). Notably, there were no significant difference in expression of the inhibitory checkpoint molecule CTLA-4 at any oxygen concentration (**Figure 3G**). Gene expression of checkpoint molecules showed a varied pattern of both activating and inhibitory molecules, with highest expression noted in FIH KO T cells (Cd28, Lag3, Tim3) or in FIH/VHL KO T cells (Icos, Ctla4, Havcr2, Pdcd1) (**Supplementary Figure 3A**). The most marked differences in the differentiation markers CD44 and CD62L occurred at 5% oxygen, with FIH loss resulting in significantly less central memory cells, and more effector memory T cells being produced (**Figures 3H, I**; **Supplementary Figure 3B**). Effector molecules, with the exception of TNF alpha, did not follow this pattern (**Figures 3J–L**).

To assess whether differences in FIH activity across oxygen concentrations may be mediated indirectly via epigenetic changes, we profiled the activity of oxygen-dependent histone lysine demethylases in T cells (**Figure 3M**). To guide selection of relevant epigenetic targets, we used publicly available datasets to profile the histone lysine demethylase expression in activated CD4+ and CD8+ T cells. We found that KDM2B/5C and KDM6A/B, which demethylate H3K4me3 and H3K27me3 marks respectively, were highly expressed early in murine T cells following activation (**Supplementary Figures 3C–E**). Although there were no significant differences expression of these genes in cells cultured at high (21%) or low (1%) levels of oxygen, (**Supplementary Figure 3F**), we found an increase in both KDM2B/5C and KDM6A/B activity in FIH null T cells, when measured as a reduction in global H3K4me3 and H3K27Me3 marks. This epigenetic change was only seen when cells were cultured at 5% oxygen, and was not seen at 21% or 1% oxygen (**Figures 3N, O**).

In summary, FIH modulates *in vitro* T cell differentiation in an oxygen-dependent manner, with maximal effects at intermediate (5%) oxygen tensions.

### FIH deletion in T cells improves *in vivo* T cell proliferation, effector differentiation and anti-tumour immunotherapy

**Figure 4:**
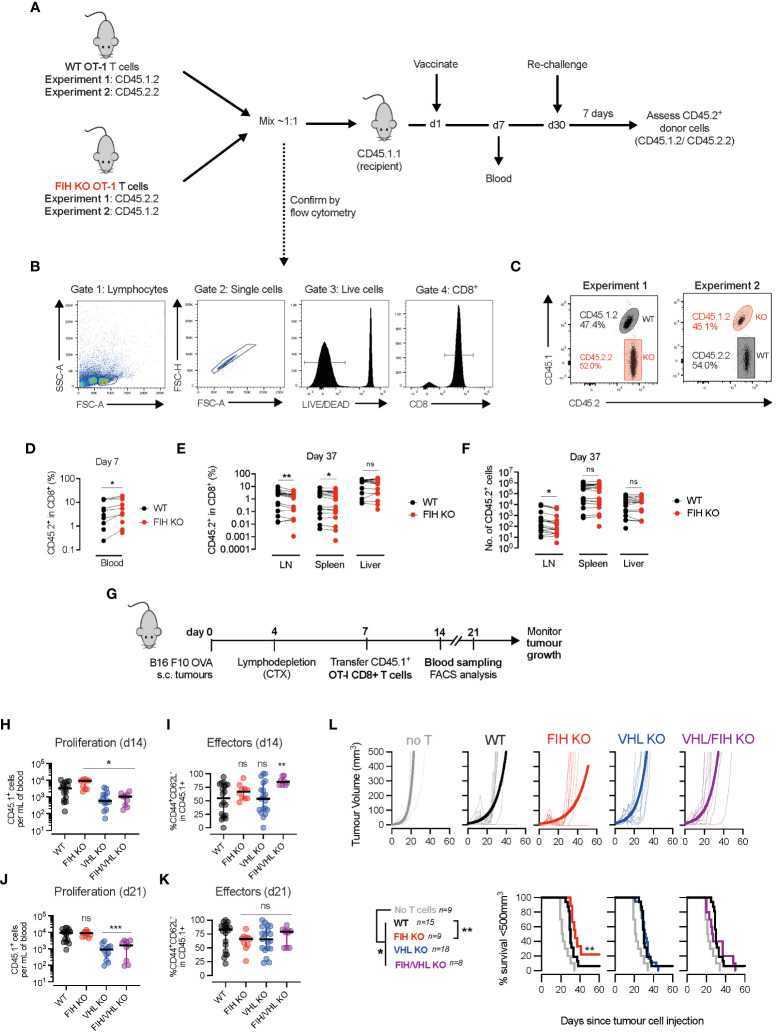
FIH loss augments *in vivo* T cell expansion and anti-tumour responses. **(A)** Timeline of two *in vivo* vaccination/recall experiments - naïve WT and FIH KO OT-I cells with distinct congenic markers (CD45.1/2.2) were mixed ~1:1 and transferred into CD45.1.1 recipient mice. The following day, mice were vaccinated with ovalbumin (OVA) peptide-loaded bone marrow derived myeloid (BMDM) cells and donor OT-I primary immune responses were measured in the blood on day 7. On day 30, mice were rechallenged with OVA peptide-loaded BMDM cells and the donor OT-I recall response was measured in secondary lymphoid organs. **(B)** Flow cytometry plots outline gating strategy to identify WT and FIH KO OT-I T cells using CD45.1/2.2 congenic markers. **(C)** Frequency of naïve WT and FIH KO OT-I cells in blood at day 0 prior measured by flow cytometry. **(D)** Frequency of WT and FIH KO OT-I cells in blood at day 7 post-transfer measured by flow cytometry (n=18 mice per genotype). **(E, F)** Frequency **(E)** and absolute numbers **(F)** of WT and FIH KO OT-I cells in blood at day 37 post-transfer measured by flow cytometry (n=18 mice per genotype). **(G)** Timeline of *in vivo* adoptive transfer immunotherapy experiment -C57BL/6 mice received a subcutaneous injection of B16 F10 OVA cells, were lymphodepleted with cyclophosphamide (CTX) on day 4 and received an intraperitoneal injection of day 3 activated WT, FIH KO, VHL KO or FIH/VHL KO CD45.1+OT-I T cells on day 7. Tumour growth was monitored over time. Expansion of CD45.1+OT-I T cells was monitored in the blood 7 and 14 days post-transfer. **(H, I)** Frequency of CD45.1+ donor cells **(H)** and CD45.1+CD44+CD62L- donor effector cells **(I)** in peripheral blood 7 days post-transfer. **(J, K)** Frequency of CD45.1+ donor cells **(J)** and CD45.1+CD44+CD62L- donor effector cells **(K)** in peripheral blood 14 days post-transfer. **(L)** Tumour volume and survival (tumour <200 mm^3^) on mice after intraperitoneal injection of PBS, WT, FIH KO, VHL KO or FIH/VHL KO OT-I T cells (n=9,15,9,18,8, respectively). Data are representative of two **(C–F)** or three **(H–L)** independent experiments. Paired t-test **(D–F)**. One-way ANOVA with Dunnett’s multiple comparison test **(H–K)**. Log-rank test **(L)**. *p<0.05, **p<0.01; ns, non-significant.

We next assessed the role of FIH in T cell function *in vivo*. We used donor WT and FIH KO OVA-specific (OT-I) T cells with distinct CD45 allelic variants (CD45.1 and/or CD45.2) to distinguish them from endogenous T cell populations in wild-type CD45.1.1+ recipients (**Figure 4A**). Equal numbers of donor WT and FIH KO OT-I T cells were transferred into recipient mice (**Figures 4B, C**) and activated *in vivo* 1 day later using OVA-pulsed bone-marrow derived macrophages (BMDM). On day 7, donor FIH KO OT-I T cells were present in greater numbers in the blood when compared to donor WT OT-I T cells (**Figure 4D**). Animals were rechallenged on day 30 to assess secondary responses. Following re-challenge, on day 37, a significantly smaller proportion of the total CD8+ population were donor FIH KO OT-I T cells, when compared to donor WT OT-I T cells in secondary lymphoid organs (lymph nodes and spleen) but not non-lymphoid organs (liver) (**Figure 4E**). Absolute numbers of donor FIH KO OT-I T cells were significantly reduced compared to WT OT-I T cells in lymph nodes but not in spleen or liver (**Figure 4F**). These *in vivo* data were in agreement with our *in vitro* data showing that FIH KO cells had lower expression of CD62L, a surface marker involved in T cell homing to secondary lymphoid organs (such as lymph nodes and spleen) (25).

To determine whether targeting FIH may be a therapeutic strategy to improve anti-tumour immune responses, we injected wild-type C57BL/6 mice with OVA-expressing melanoma (B16 F10 OVA) tumours, and following lymphodepletion, treated them with either PBS (control) or activated WT, FIH KO, VHL KO or FIH/VHL KO OT-I T cells (**Figure 4G**, **Supplementary Figure 4A**). On day 14, 1 week following T cell transfer, we found that when compared to WT OT-I cells, there was a significantly increased number of circulating FIH KO OT-I T cells, and a significantly lower number of circulating VHL KO or FIH/VHL KO OT-I T cells (**Figure 4H**). When compared to WT OT-I cells, only FIH/VHL KO OT-I T cells had a significantly higher proportion of CD44+CD62L- effector cells (**Figure 4I**). By day 21, 2 weeks following T cell transfer, the number of circulating FIH KO OT-I T cells was similar compared to WT OT-I cells, and there continued to be a significantly lower number of circulating cells in groups lacking VHL (VHL KO and FIH/VHL KO) (**Figure 4J**). There were no significant differences in the proportion of effector cells across genotypes at day 21 (**Figure 4K**).

To assess anti-tumour activity of T cell populations of each genotype we measured tumour volumes over time. Wild-type tumour-bearing mice that received T cells of any genotype had significantly improved tumour control when compared to tumour-bearing mice that received no T cells. However, only mice treated with FIH KO OT-I T cells exhibited significantly improved tumour control, whereas VHL KO OT-I and FIH/VHL KO OT-I T cells resulted in equivalent anti-tumour control when compared to WT OT-I T cell responses (**Figure 4L**). No significant differences in tumour growth were noted in mice that had T cell-specific deletions of FIH (**Supplementary Figures 4B, C**) or in orthotopic or adoptive cell transfer models using tumour cells lacking FIH (**Supplementary Figures 4D–H**).

## Discussion

We show here that FIH regulates HIF activity in T cells and alters T cell function in a HIF-dependent manner. Our data indicates that FIH has maximal effects at intermediate oxygen levels, and that FIH activity can indirectly modulate the activity of other oxygen-dependent enzymes. In addition, we found that loss of FIH alone in T cells improves *in vivo* expansion, effector differentiation and anti-tumour activity in an adoptive cell immunotherapy model.

We profiled FIH activity across a range of substrate/co-substrate levels - by varying oxygen levels and by using concomitant VHL deletion to achieve constitutively-high HIF levels – to capture dynamic aspects of FIH activity. Our results suggest that FIH operates maximally at intermediate (5%) oxygen levels, with increases or decreases in oxygen tensions resulting in a reduced overall effect size when comparing WT with FIH KO T cells. We found that increasing HIF activity by FIH deletion boosts effector differentiation and anti-tumour activity, but further increasing HIF activity, through combined FIH and VHL deletion, results in suppression of proliferation, reduced interferon-gamma production, and removes the *in vivo* anti-tumour advantage conferred by FIH KO T cells. This Goldilocks principle for HIF function in the T cell - not too much, not too little - has both physiological significance in defining the substrate/co-substrate conditions under which FIH is active, but also functional relevance in guiding the targeting of HIF activity in T cells to optimise anti-tumour immunotherapy.

Our finding that FIH loss alone can boost anti-tumour immunotherapy is in agreement with our own prior work on overexpression of HIF1 and HIF2 isoforms with differential sensitivity to FIH and VHL control (9). In those studies, only overexpression of FIH-insensitive HIF2a in T cells resulted in improved anti-tumour control; VHL- and FIH-insensitive HIF2 in T cells circulate in the periphery to a lesser extent and do not significantly improve anti-tumour control when compared to T cells transduced with a control vector (9). Our group and others have assessed the role of VHL loss in T cell-mediated anti-viral and anti-tumour activity (10, 26, 27). In our present study we show that VHL knockout T cells have reduced peripheral circulation over time and show no significant improvement in anti-tumour function compared to wild-type T cells This is in contrast to previously published studies that demonstrated improved anti-tumour control with VHL KO T cells (10, 27). Notably, these prior studies adoptively transferred 6 times the number of T cells we used here, (3 million versus 0.5 million) into melanoma-bearing mice; this may have resulted in the delivery of a greater number of cells to the tumour site. Since the VHL ko cells do show increased expression of some cytotoxic effector markers relative to WT T cells, this coupled to an initially increased T cell number may account for the differences in anti-tumour responses noted between these studies.

To assess whether the anti-tumour activity of FIH KO T cells was a restricted or more general feature, we carried out *in vivo* experiments using an adoptive transfer model, with MHC-I restricted OT-I T cells transferred into B16 tumour-bearing mice, as well as an orthotopic B16 tumour model using wild-type or T cell-specific FIH knockout mice. We noted improved tumour control with FIH knockout OT-I T cells in the adoptive transfer model, but no significant differences in tumour control between wild-type or T cell-specific FIH knockout mice in an orthotopic model. Based on these findings, we conjecture that FIH loss may improve T cell function in adoptive transfer due to ex vivo pre-transfer conditioning, or because it utilises a restricted population of CD8+ T cells. In addition, in contrast to selecting a purified population of MHC-I restricted OT-I cells, the FIH knockout is present in all T cell subsets in the orthotopic model, with different contributions from effector (e.g., CD8+ T cells) and suppressor populations (e.g., CD4+ regulatory T cells); these other cell types may thus be influencing overall anti-tumour outcomes.

FIH responses likely vary across cell types and tissues depending on location-specific substrate/co-substrate (HIF and oxygen) levels. In activated T cells cultured at 21% oxygen, we found that loss of FIH suppressed oxygen consumption, in keeping with disinhibited HIF activity. Whereas, as demonstrated in previous work from our group, FIH loss in muscle cells results in increased oxygen consumption; only in FIH/VHL double knockout muscle cells is oxygen consumption again suppressed (18). These differences in substrate/co-substrate availability and utilisation, and consequently FIH activity, may be a feature that is tuned to the requirements of each specific cell or tissue type. For example, T cells must rapidly engage HIF-driven metabolism to proliferate following activation, whereas predominantly non-proliferating muscle cells utilise FIH to dynamically control oxygen utilisation and to optimise their function (18). Furthermore, cell-specific regulation of the HIF transcriptional axis – such as increases in HIF1 levels in activated T cells secondary to TCR-induced increases in *HIF1a* mRNA transcription and translation – likely provide a further layer of modulation of FIH activity.

In addition to regulating HIF-driven transcriptional activity, FIH may mediate its effects by indirectly altering the activity of oxygen-dependent enzymes. We show that the activity of the oxygen-dependent enzymes Kdm2b/5a and Kdm6a/b (which demethylate H3K4me3 and H3K27me3, respectively, during T cell differentiation) changes in an FIH- and oxygen-dependent manner. This epigenetic reprogramming may account for differences in T cell differentiation noted between wild-type and FIH knockout T cells at different levels of HIF and oxygen. Our findings are consistent with previous studies that highlight the roles of Kdm5a and Kdm6a as oxygen-sensors and regulators of cell differentiation (28, 29), suggesting FIH as an additional regulator of these epigenetic changes, particularly at intermediate (5%) oxygen tensions. These studies, establishing the role of FIH in an important immune population, illustrate the potential for utilising the HIF pathway to alter and ultimately enhance immune function in immunotherapy and other important immunological applications.

## Methods

### Animals

Animal work was carried out under UK Home Office guidelines and the approval of the Regional Animal Ethics Committee of Northern Stockholm, Sweden. Mice were housed in a specific pathogen-free animal facility, provided with food and water *ad libitum*, and maintained on a 12 hours light-dark cycle at 21°C. Genotyping was performed with DNA from ear biopsies using commercial Transnetyx qPCR assays. C57Bl/6J (CD45.2) mice were purchased from Janvier Labs. Mice with loxP sites flanking exon 2 of the mouse FIH gene (*Hif1an* ^fl/fl^) (17) were crossed with *dLck* Cre mice to efficiently delete the floxed FIH allele in CD8+ T cells. *Vhl* ^fl/fl^ *dLck* Cre mice were generated as previously described^9^ and crossed with *Hif1an* ^fl/fl^ *dLck* Cre mice to make *Hif1an* ^fl/fl^ *Vhl* ^fl/fl^ *dLck* Cre mice. Donor TCR transgenic OT-I+ mice (JAX 003831, The Jackson Laboratory) were crossed with mice bearing the CD45.1 congenic marker (JAX 002014, The Jackson Laboratory). These OT-I+CD45.1+ mice were then crossed with FIH dLck mice. All mice were backcrossed over ten generations to the C57BL/6J background. Male and female animals >6 weeks of age were used in experiments. Groups were assigned randomly for mouse experiments and the investigator was blinded to group assignment. No statistical methods were used to pre-determine sample size.

### Cell lines

B16 F10 (mouse melanoma) cells were purchased from ATCC (CRL-6475). Ovalbumin-expressing B16 F10 (B16 F10 OVA) cells were generated in-house by Dr Pedro Velica by co-transfection of the transposon vector pT2 encoding OVA, eGFP and neomycin phosphotransferase and the vector encoding transposase SB11. After three days, 400 μg/ml G418 (Gibco, 10131035) was added to culture media to select for transgene-expressing cells. Successful integration was confirmed by analysing eGFP fluorescence by flow cytometry. Limiting dilution was used to derive monoclonal OVA-expressing lines for each cell line. OVA presentation was confirmed by flow cytometry using a PE-labelled antibody against surface SIINFEKL bound to H-2Kb (clone 25-D1.16, BioLegend).

### Cell culture

B16 F10 OVA cells were grown in T75 cell culture flasks (Sarstedt) in high-glucose DMEM supplemented with 10% fetal bovine serum (FBS), 1% penicillin/streptomycin and 0.75mg/ml G418 (all Thermo Fisher). Mouse CD8+ and OT-I T cells were grown in 48 well plates (Corning) in RPMI media supplemented with 10% fetal bovine serum (FBS), 1% penicillin/streptomycin, 55μM 2-ME (all Thermo Fisher) and 100 units/ml recombinant human IL-2 (Roche). Cells were cultured in humidified incubators with 5% carbon dioxide and 1%, 5% or 21% oxygen, as indicated in figure legends.

### Cell count and viability measurement

Cells were either counted using an automated cell counter (BioRad TC20) with viability assessed by trypan blue exclusion or were stained with LIVE/DEAD (Life Technologies) viability dye and absolute numbers were determined by flow cytometry using counting beads (Countbright, Life Technologies).

### Cell division analysis

Cells were washed in PBS and incubated with 5μM CellTrace Violet (Invitrogen C34557) in PBS for 30 minutes. RPMI media was then added for 5 minutes, after which the cells underwent centrifugation and were resuspended in fresh RPMI for onward culture. In synchronous populations (e.g. activated T cells), cell division analysis was carried out by comparing division number, whereas with asynchronous populations (e.g. EL4 cell line) samples were compared using mean fluorescence intensity.

### T cell isolation and activation

CD8+ T cells were isolated from mouse spleens and purified using Microbeads conjugated to monoclonal anti-mouse CD8α (Ly-2; isotype: rat IgG2a) antibody (Miltenyi, 130-117-044), followed by magnetic bead isolation on a MACS column. CD8+ T cells were activated with anti-mouse CD3/CD28 Dynabeads (Thermo Fisher) at a 1:1 cell/bead ratio. After 3 days of culture, Dynabeads were removed using a magnet and cells were prepared for flow cytometry analysis or cultured for a further 4 days. OT-I CD8+ T cells from OT-I mouse spleens were activated with anti-mouse CD3/CD28 Dynabeads or with 1μg/ml SIINFEKL peptide for 48 hours. Following this, OT-I T cells were washed twice with PBS and cultured for a further 5 days. All T cells were cultured in complete RPMI medium containing 2mM glutamine, supplemented with 10% FBS, 1% Streptomycin, 55μM β-mercaptoethanol (all Thermo Fisher) and 100 units/ml recombinant human IL-2 (Roche, 10 799 068 001).

### Baseline lymphoid tissue phenotyping

Female *Hif1an* ^fl/fl^ *dLck* Cre- (‘WT’) and *Hif1an* ^fl/fl^ *dLck* Cre+ (‘FIH KO’) mice, aged between 7 - 12 weeks, were sacrificed and lymph nodes, spleen and liver were harvested. Single cell suspensions were created by passing each organ through a 40 μM strainer (VWR). Cells were incubated with Fc block (Biolegend) followed by surface antibody staining, as outlined previously. Samples were analysed using a FACSCanto II flow cytometer (BD Biosciences) and counting beads were used to quantify absolute cell numbers (CountBright, Life Technologies).

### qRT-PCR

Total RNA was extracted from isolated CD8+ T cells (RNeasy kit, Qiagen) and 300 ng of RNA were used for cDNA synthesis (First-Strand Synthesis kit, Invitrogen). All kits were used according to the manufacturer’s instructions. Samples were run in technical duplicates. Quantitative real time PCR (qPCR) was performed in a StepOnePlus system (Applied Biosystems) with 10 µL reactions composed of 4µL cDNA (10x diluted), 1µL primer solution (3 µM) and 5 µL FastStart Universal SYBR Green Master (4913914001, Roche). *Hprt* was used as a housekeeping gene. All primers were acquired from Integrated DNA Technologies (Predesigned qPCR assays). Data were analysed by the ddCt method by normalising the expression of each gene for each replicate to Hprt and then to the WT control group.

### Immunoblotting

Nuclear and cytoplasmic extracts from CD8^+^ T cells were obtained with the NE-PER Nuclear and Cytoplasmic Extraction Reagents (78833, ThermoFisher). Proteins (10-20 µg) were separated by SDS-PAGE and transferred to nitrocellulose or PVDF membranes which were incubated for 1 hour at room temperature with a blocking solution (1X ROTI Block (A151.4, Roth) or 5% non-fat milk in PBS 0.1% Tween-20). Then, membranes were incubated in 50 mL tubes with a 3 mL of blocking solution containing the antibodies. Primary antibodies were used at a 1:1,000 dilution and incubated overnight at 4°C in a tube roller and secondary antibodies were used at a 1:5,000 dilution and incubated for 2 hours at room temperature in a tube roller. Protein extracts were probed with primary antibodies against FIH (Santa Cruz sc-271780), Tubulin (Abcam ab6160), HIF-1a (Novus NB-100–449), Histone 3 (CST 4499), and HDAC (Abcam ab109411) and detected using the secondary antibody anti-rabbit (926–32213, LI-COR), anti-mouse (926–68070, LI-COR) or anti-rabbit IgG (R&D HAF008). After each antibody probing, membranes were washed 3x10 minutes in the 50 mL tubes with 10 mL of blocking solution. Protein signal was detected using infra-red labelled secondary antibodies in an Odyssey imaging system (LI-COR) or using horseradish peroxidase-conjugated secondary antibody (R&D systems) and ECL Prime (GE Healthcare) imaged with an iBrightCL1000 (ThermoFisher).

### CRISPR/Cas9 knockout of FIH

To achieve a CRISPR/Cas9-mediated gene knockout of FIH in B16 F10 melanoma cells, Dr Pedro Veliça designed three gRNAs targeting exon 1 of mouse FIH (*Hif1an*):

gRNA#1 GGCCGTCCCTAGAGTAGAGATGG, gRNA#2:GGCGACGGCAGCCGAGGTTGCGG, gRNA#3:GGAAGAGCTCATCGAAAATGAGG, and cloned them into pX330-U6-Chimeric_BB-CBh-hSpCas9 (#42230, Addgene). B16F10-OVA cells were transfected with the three vectors FuGENE HD Transfection Reagent, following the manufacturer’s instructions. Cell clones were obtained through limiting dilution. Immunoblotting was used to identify clones with successful FIH knockout.

### *In vivo* orthotopic tumour model

Female *Hif1an* ^fl/fl^ *dLck* Cre- (‘WT’) and *Hif1an* ^fl/fl^ *dLck* Cre+ (‘FIH KO’) mice, aged between 7 - 12 weeks, were injected subcutaneously with 5 x 10^5^ B16 F10 OVA and tumour growth was monitored over time until a humane endpoint was reached. Animals were assigned randomly to each experimental group. Tumours were measured every 2-3 days (daily evaluations during injections) with digital callipers. Tumour volume was calculated using the formula: 1/2 (a^2^ x b), where a is the width and b is the length of the tumour.

### FIH knockout tumour model

Female C57Bl/6 mice aged between 7 - 12 weeks, were injected subcutaneously with 5 x 10^5^ of WT or FIH KO B16 F10 OVA cells and tumour growth was monitored over time until a humane endpoint was reached. Animals were assigned randomly to each experimental group. Tumours were measured every 2-3 days (daily evaluations during injections) with electronic callipers. Tumour volume was calculated using the formula: 1/2 (a^2^ x b), where a is the width and b is the length of the tumour.

### *In vivo* proliferation and recall experiments

Splenocytes from OT-I CD45.1.1. and CD45.1.2 from *Hif1an* ^fl/fl^ *dLck* Cre- (‘WT’) and *Hif1an* ^fl/fl^ *dLck* Cre+ (‘FIH KO’) mice and naive CD8+ T cells were purified using magnetic negative selection beads (Miltenyi). Six days prior to this, bone marrow-derived myeloid (BMDM) cells were extracted from wild-type C57BL/6J mice and cultured in non-TC treated culture dishes in high-glucose DMEM (10% FBS, 1% penicillin-streptomycin) supplemented with 10 ng/ml GM-CSF and 10 ng/ml M-CSF. After 7 days of culturing, BMDM cells were activated with 100 ng/ml LPS (Sigma) for 24 hours, then detached from plates using Cell Lifters (Corning) and CellStripper non-enzymatic dissociation solution (Corning) and loaded with 100 ng/ml SIINFEKL peptide at 37°C for 1 hour. Naive WT and FIH KO OT-I CD8+ T cells were washed with PBS, mixed 1:1 and a total of 1 million cells (0.5 million per group) and were injected intraperitoneally into C57BL/6J wild-type CD45.1.1 recipient mice. The following day, a total of 1 million SIINFEKL-loaded BMDM cells were washed with PBS and injected intraperitoneally into C57BL/6J wild-type CD45.1.1 recipient mice. Peripheral blood was collected from the tail vein of CD45.1.1 recipient mice 7 days after T cell transfer to confirm T cell activation and expansion. After 30 days, BMDM cells, prepared as described above, were injected intraperitoneally into CD45.1.1 recipient mice to generate a recall response. After a further 7 days, mice were sacrificed and lymph nodes, spleen and liver were harvested and analysed by flow cytometry. Absolute numbers of cells were determined using counting beads (CountBright, Life Technologies).

### *In vivo* tumour adoptive immunotherapy model

Magnetic negative selection beads (Miltenyi) were used to isolate CD8+ T cells from spleens from *Vhl* ^fl/fl^ *dLck* Cre- (‘WT’), *Hif1an* ^fl/fl^ *dLck* Cre+ (‘FIH KO’), *Vhl* ^fl/fl^ *dLck* Cre+ (‘VHL KO’) and *Hif1an* ^fl/fl^ *Vhl* ^fl/fl^ *dLck* Cre+ (‘FIH/VHL KO’) OT-I mice. CD8+ T cells were then activated with anti-CD3/CD28 Dynabeads and cultured for 3 days in the presence of IL-2. On day 7, 0.5 x 10^6^ OT-I cells were transferred intraperitoneally into tumour-bearing C57BL/6J mice that had undergone lymphodepletion 3 days prior by intraperitoneal injection of 300 mg/kg cyclophosphamide. The expansion of OT-I T cells in the blood was evaluated from tail vein blood samples taken on day 14 and day 21 (7 days and 14 days following OT-I transfer, respectively). Tumour growth was evaluated until a humane endpoint was reached. Tumours were measured blindly every 2-3 days (daily evaluations during injections) with digital callipers and tumour volume was calculated using the formula: (*a* ^2^ x *b*) x 0.5 where *a* is the width and *b* is the length of the tumour.

### Flow cytometry

Single cell suspensions were stained with Near-IR Dead Cell Stain Kit (ThermoFisher) followed by surface, cytoplasmic and/or nuclear staining with the following fluorochrome-conjugated monoclonal antibodies: CD25 (PC61.5) purchased from ThermoFisher; H3K4me3 (C42D8), H3K27me3 (C3B11) purchased from Cell Signaling Technology; 4-1BB/CD137 (1AH2), TNF-α (MP6-XT22) purchased from BD Biosciences; CD44 (IM7), CD62L (MEL-14), CD8 (53-6.7), Fc Block (93), Granzyme B (QA16A02), ICOS (C398.4A), IFN-γ (XMG1.2) purchased from Biolegend. Staining of cytoplasmic and nuclear antigens was performed using the Fixation/Permeabilisation kit (BD Biosciences) and the Transcription Factor buffer set (Invitrogen/eBiosciences), respectively. To measure intracellular cytokines, cells were re-stimulated with PMA, Ionomycin and Brefeldin (all Sigma) for 4 hours prior to staining. Samples were analysed on a FACSCanto II flow cytometer (BD Biosciences).

### *In vitro* cytotoxicity assay

To assess T cell cytotoxicity, 10,000 B16 F10 OVA cells were plated in a flat-bottom 96 well plate (Costar) and allowed to adhere for 4 hours before addition of 1e2-1e6 WT, FIH KO, VHL KO or FIH/VHL KO OT-I T cells, to achieve final effector:target (E:T) ratios ranging from 100:1 - 1:100. Following 20 hours of co-culture in 21, 5 or 1% oxygen, wells were washed three times with PBS to remove T cells and the remaining target cells were determined by culturing with 10 μg/ml resazurin (Sigma) in RPMI media for 2 hours and measuring fluorescence signal with a plate reader. Cytotoxicity was calculated relative to wells with no T cells added (positive control) and to wells with no B16 F10 OVA cells added (negative control).

### RNA-seq

CD8+ T cells were purified from *Hif1an* ^fl/fl^ *dLck* Cre- or *Vhl* ^fl/fl^ *dLck* Cre- (‘WT’), *Hif1an* ^fl/fl^ *dLck* Cre+ (‘FIH KO’), *Vhl* ^fl/fl^ *dLck* Cre+ (‘VHL KO’) and *Hif1an* ^fl/fl^ *Vhl* ^fl/fl^ *dLck* Cre+ (‘FIH/VHL KO’) mice spleens using Microbeads conjugated to monoclonal anti-mouse CD8α (Ly-2; isotype: rat IgG2a) antibody (Miltenyi, 130-117-044), followed by magnetic bead isolation on a MACS column. CD8+ T cells were activated with anti-mouse CD3/CD28 Dynabeads (Thermo Fisher) at a 1:1 cell/bead ratio. After 3 days of culture, Dynabeads were removed using a magnet and cells were washed twice with ice cold PBS and lysed with RLT buffer containing 1% β-mercaptoethanol. Total RNA was subjected to quality control with Agilent Tapestation according to the manufacturer’s instructions. To construct libraries suitable for Illumina sequencing the Illumina TruSeq Stranded mRNA Sample preparation protocol which includes cDNA synthesis, ligation of adapters and amplification of indexed libraries was used. The yield and quality of the amplified libraries were analysed using Qubit by Thermo Fisher and the Agilent Tapestation. The indexed cDNA libraries were normalised and combined and the pools were sequenced on the Nextseq 550 for a 50-cycle v2.5 sequencing run generating 2x75 bp paired-end reads. The raw RNA-seq data are deposited in the GEO database under the series number GSE234381.

### RNA-seq data analysis

Base-calling and demultiplexing was performed using CASAVA software with default settings generating Fastq files for further downstream mapping and analysis. Sequenced reads were mapped to the genome using the STAR aligner with default settings and uniquely mapped reads were counted. Normalised counts and differential gene expression were determined with DESeq2 (30). Principal component analysis (PCA) was carried out using the R *prcomp* function. Heatmaps were generated using the R *pheatmap* package. Hierarchical clustering was carried out using the R *hclust* function. Pathway enrichment analysis of gene clusters or significantly differentially-expressed (FDR threshold 5%) gene sets were carried out using the *enricher* function from the R ClusterProfiler (v.4.6.2) package using Hallmark gene sets from the Molecular Signature Database (MSigDB (31)). HIF1 target genes were obtained from the Semenza HIF1 target geneset (22), available as Hallmark geneset. Resulting data were visualised using functions available in the R ClusterProfiler and ggplot2 (v.3.4.2) packages. All calculations, including Z scores and log fold changes were performed in R version 4.2.3.

### Seahorse

Oxygen consumption rates (OCR) and extracellular acidification rates (ECAR) were measured on a Seahorse XFe96 Analyzer (Agilent) using 2 x10^5^ day 3 activated CD8+ T cells per well on a poly-D-lysine coated plate with XF RPMI media pH 7.4 (containing 10 mM glucose, 2 mM L-glutamine). Measurements were made under basal conditions and following addition of 1 μM oligomycin, 1.5 μM FCCP or 0.1 μM rotenone and 1 μM antimycin. All compounds were acquired from Sigma-Aldrich.

### Statistical analysis

Statistical analyses were performed in GraphPad Prism 9 software and R version 4.2.3. Pairwise comparisons of unpaired data were carried out using a two-tailed Student’s t-test with Welch’s correction, where appropriate. Pairwise comparison of paired data was carried out using a paired t-test. Multiple comparisons were carried out using one-way ANOVA with Dunnett’s multiple comparison test or using multiple t-tests with multiple comparison correction via the two-stage step-up method of Benjamini, Krieger and Yekutieli. Grouped data was assessed by two-way ANOVA, correcting with Šídák’s multiple comparisons test. Error bars shown represent s.e.m., unless otherwise stated in figure legends. Sample sizes were chosen based on previous experience of *in vitro* and *in vivo* experiments.

### Experimental samples

Animals and cell samples used in this work have not been utilised in other manuscripts.

## Data availability statement

The datasets presented in this study can be found in online repositories. The names of the repository/repositories and accession number(s) can be found below: GEO database under the series number GSE234381.

## Ethics statement

The animal study was approved by Regional Animal Ethics Committee of Northern Stockholm, Sweden. The study was conducted in accordance with the local legislation and institutional requirements.

## Author contributions

RSJ: Conceptualization, Funding acquisition, Project administration, Supervision, Writing – original draft, Writing – review & editing. DB: Conceptualization, Formal analysis, Investigation, Methodology, Resources, Writing – original draft, Writing – review & editing. PC: Conceptualization, Formal analysis, Investigation, Methodology, Writing – original draft, Writing – review & editing. PV: Conceptualization, Formal analysis, Investigation, Writing – review & editing. LK: Investigation, Writing – review & editing. MB: Investigation, Writing – review & editing. LB: Investigation, Writing – review & editing. MG: Investigation, Writing – review & editing. IF: Investigation, Project administration, Writing – review & editing. HR: Project administration, Supervision, Writing – review & editing.
