# Supplementary material for: The factor inhibiting HIF regulates T cell differentiation and anti-tumour efficacy
Source: Front Immunol. 2024 Apr 16;15:1293723. doi: 10.3389/fimmu.2024.1293723 (PMC11058823; doi:10.3389/fimmu.2024.1293723)
Supplement: Supplementary file 1 [file Image_1.pdf]

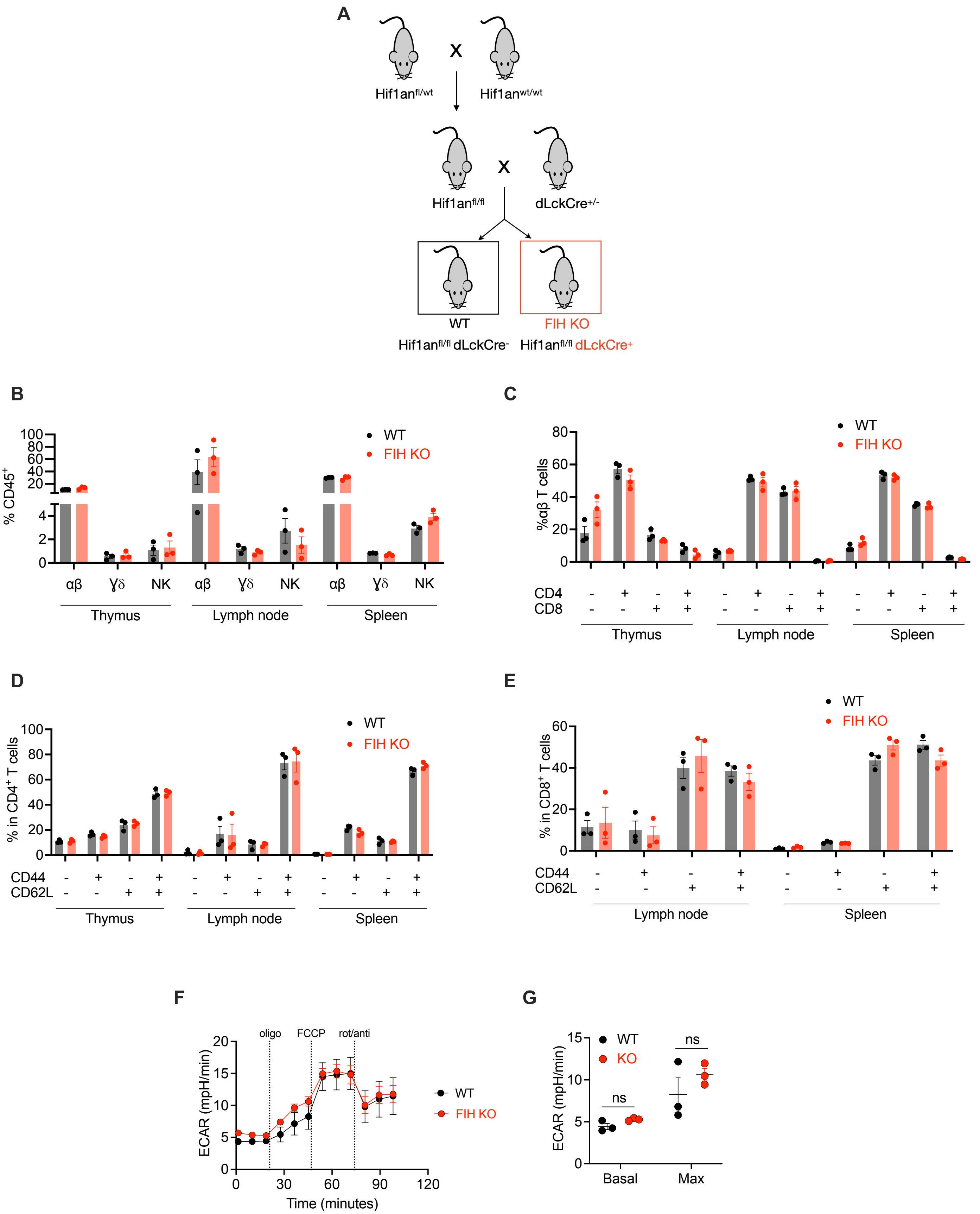
A
Hif1an fl/wt
X
Hif1an wt/wt
Hif1an fl/fl
dLckCre+/-
WT
Hif1an fl/fl dLckCre-
FIH KO
Hif1an fl/fl dLckCre+
B
% CD45+
WT
FIH KO
αβ
γδ
NK
Thymus
Lymph node
Spleen
C
%αβ T cells
CD4
CD8
D
% in CD4+ T cells
CD44
CD62L
E
% in CD8+ T cells
F
oligo
FCCP
rot/anti
ECAR (mpH/min)
Time (minutes)
G
KO
ns
Basal
Max

Supplementary Figure 1: Loss of FIH does not significantly alter T cell development in mice. (A) Schematic illustrating mouse crosses to generate T cell-specific FIH knockout. FIH expression levels from publicly-available mouse OT-I T cell mRNA (**(B)**, Immgen) and mouse CD8+ T cell protein (**(C)**, IMPRESS) expression data. **(D)** Frequency of αβ-T cells, ɣδ-T cells and NK cells as a proportion of total CD45+ cells in thymus, lymph nodes and spleens of 8-week-old *Hif1an*$^{fl/fl}$ *dLck* Cre negative ('WT') and *Hif1an*$^{fl/fl}$ *dLck* Cre positive ('FIH KO') mice (n=3 mice per genotype). **(E)** Frequency of CD4-CD8-, CD4+CD8-, CD4-CD8+and CD4+CD8+ cells as a proportion of total αβ T cells in thymus, lymph nodes and spleens of 8-week-old WT and FIH KO mice (n=3 mice per genotype). **(F,G)** Frequency of CD44-CD62L-, CD44+CD62L-, CD44-CD62L+ and CD44+CD62L+ cells as a proportion of total CD4+ T cells (d) or CD8+ T cells (e) in thymus, lymph nodes and spleens of 8-week-old WT and FIH KO mice (n=3 mice per genotype). **(H)** Seahorse metabolic profile showing extracellular acidification rate of activated WT and FIH KO CD8+ T cells (n=3 mice per genotype). **(I)** Basal and maximal extracellular acidification rates in activated WT and FIH KO CD8+T cells (n=3 mice per genotype). Error bars denote s.e.m or s.d **(C)**. One-way ANOVA with Dunnett's multiple comparisons test **(B)**. Multiple unpaired t tests with Šídák's multiple comparison test **(D-G)**. Two-way ANOVA with Šídák's multiple comparisons test **(I)**. ns, non-significant. ECAR, extracellular acidification rate.

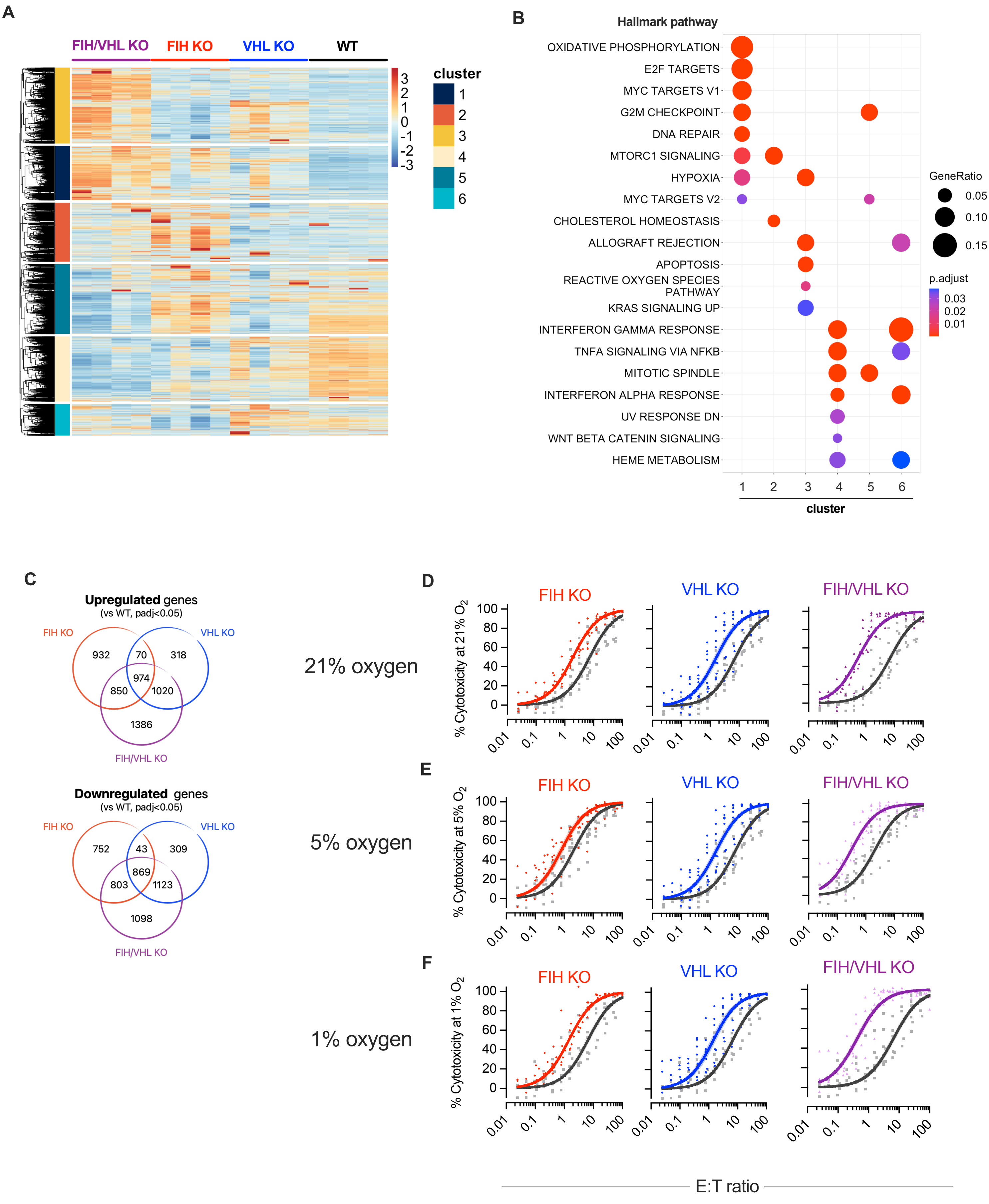
A
FIH/VHL KO
FIH KO
VHL KO
WT
cluster
1
2
3
4
5
6
3
2
1
0
-1
-2
-3
B
Hallmark pathway
OXIDATIVE PHOSPHORYLATION
E2F TARGETS
MYC TARGETS V1
G2M CHECKPOINT
DNA REPAIR
MTORC1 SIGNALING
HYPOXIA
MYC TARGETS V2
CHOLESTEROL HOMEOSTASIS
ALLOGRAFT REJECTION
APOPTOSIS
REACTIVE OXYGEN SPECIES PATHWAY
KRAS SIGNALING UP
INTERFERON GAMMA RESPONSE
TNFA SIGNALING VIA NFKB
MITOTIC SPINDLE
INTERFERON ALPHA RESPONSE
UV RESPONSE DN
WNT BETA CATENIN SIGNALING
HEME METABOLISM
GeneRatio
0.05
0.10
0.15
p.adjust
0.03
0.02
0.01
1 2 3 4 5 6
cluster
C
Upregulated genes
(vs WT, padj<0.05)
FIH KO
VHL KO
932
70
318
974
850
1020
1386
FIH/VHL KO
Downregulated genes
(vs WT, padj<0.05)
FIH KO
VHL KO
752
43
309
869
803
1123
1098
FIH/VHL KO
21% oxygen
5% oxygen
1% oxygen
D
E
F
FIH KO
VHL KO
FIH/VHL KO
% Cytotoxicity at 21% O_2
% Cytotoxicity at 5% O_2
% Cytotoxicity at 1% O_2
0
20
40
60
80
100
0.01
0.1
1
10
100
E:T ratio

Supplemental Figure 2: FIH loss increases *in vitro* T cell cytotoxicity. (A) Heatmap showing clustered RNAseq gene expression (Z score) values from activated WT (black), FIH KO (red), VHL KO (blue) and FIH/VHL (purple) CD8+ T cells (n=4 mice per genotype). **(B)** Pathway enrichment analysis using mSigDB Hallmark dataset to evaluate gene expressed in each cluster from (A). **(C)** Venn diagrams showing number of overlapping significantly upregulated/downregulated genes when comparing FIH KO, VHL KO or FIH/VHL CD8+ T cells to WT cells. **(D-F)** *In vitro* cytotoxicity assay of WT (black), FIH KO (red), VHL KO (blue) and FIH/VHL (purple) CD8+ T cells with B16 F10 OVA cells at 21% **(D)**, 5% **(E)** and 1% **(F)** oxygen across a range of effector to target (E:T) ratios.

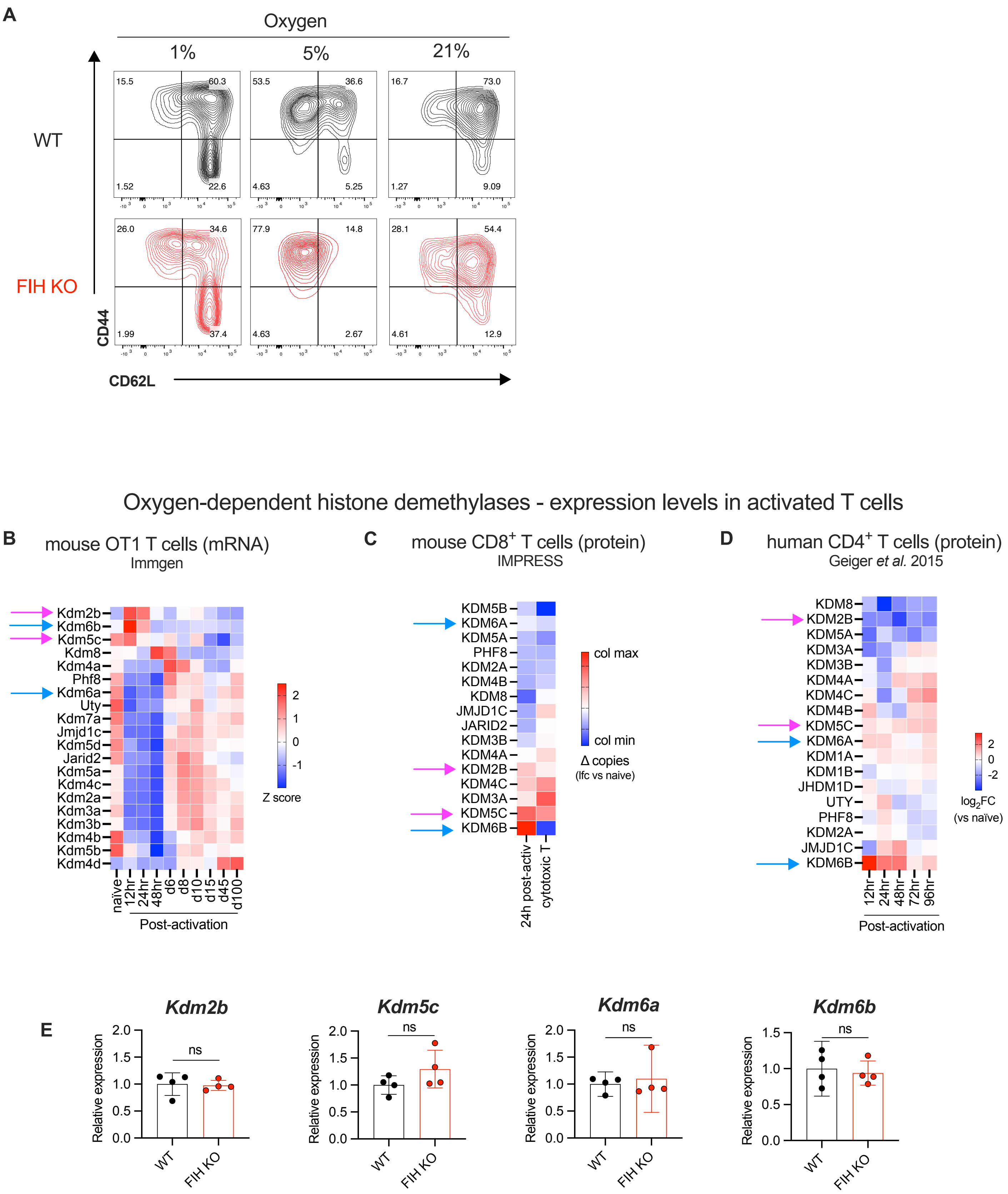
A
Oxygen
1%
5%
21%
WT
FIH KO
CD44
CD62L
15.5
60.3
1.52
22.6
53.5
36.6
4.63
5.25
16.7
73.0
1.27
9.09
26.0
34.6
1.99
37.4
77.9
14.8
4.63
2.67
28.1
54.4
4.61
12.9
Oxygen-dependent histone demethylases - expression levels in activated T cells
B
mouse OT1 T cells (mRNA)
Immgen
Kdm2b
Kdm6b
Kdm5c
Kdm8
Kdm4a
Phf8
Kdm6a
Uty
Kdm7a
Jmjd1c
Kdm5d
Jarid2
Kdm5a
Kdm4c
Kdm2a
Kdm3a
Kdm3b
Kdm4b
Kdm5b
Kdm4d
naïve
12hr
24hr
48hr
d6
d8
d10
d15
d45
d100
Post-activation
2
1
0
-1
Z score
C
mouse CD8+ T cells (protein)
IMPRESS
KDM5B
KDM6A
KDM5A
PHF8
KDM2A
KDM4B
KDM8
JMJD1C
JARID2
KDM3B
KDM4A
KDM2B
KDM4C
KDM3A
KDM5C
KDM6B
24h post-activ
cytotoxic T
col max
col min
Δ copies
(lfc vs naive)
D
human CD4+ T cells (protein)
Geiger et al. 2015
KDM8
KDM2B
KDM5A
KDM3A
KDM3B
KDM4A
KDM4C
KDM4B
KDM5C
KDM6A
KDM1A
KDM1B
JHDM1D
UTY
PHF8
KDM2A
JMJD1C
KDM6B
12hr
24hr
48hr
72hr
96hr
Post-activation
2
0
-2
log2FC
(vs naïve)
E
Kdm2b
Kdm5c
Kdm6a
Kdm6b
Relative expression
ns
WT
FIH KO
0.0
0.5
1.0
1.5
2.0

Supplementary Figure 3: Oxygen-dependent enzymes as modulators of T cell fate. (A) Z score gene expression of checkpoint molecule genes in WT, FIH KO, VHL KO or FIH/VHL CD8+ T cells (n=4 mice per genotype). **(B)** Flow cytometry plots of CD44/CD62L marker expression on activated WT and FIH KO mouse CD8+ T cells cultured at 21%, 5% and 1% oxygen for 3 days. **(C-E)** Expression levels of oxygen-dependent histone demethylases in publicly-available mouse OT-I T cell mRNA (**(C)**, Immgen), mouse CD8+ T cell protein (**(D)**, IMPRESS) and human CD4+ T cell protein (**(E)**, Geiger *et al.* 2015) datasets. **(F)** Normalized gene expression of Kdm2b, Kdm5c, Kdm6a and Kdm6b from RNAseq of day 3 activated WT and FIH KO CD8+ T cells (n=4 mice per genotype). Pink arrows highlighting KDM2B/5C levels, blue arrows highlighting KDM6A/6B levels. Error bars denote s.e.m. Unpaired t test **(F)**. ns, non-significant. FC, fold change; lfc, log_2 fold change.

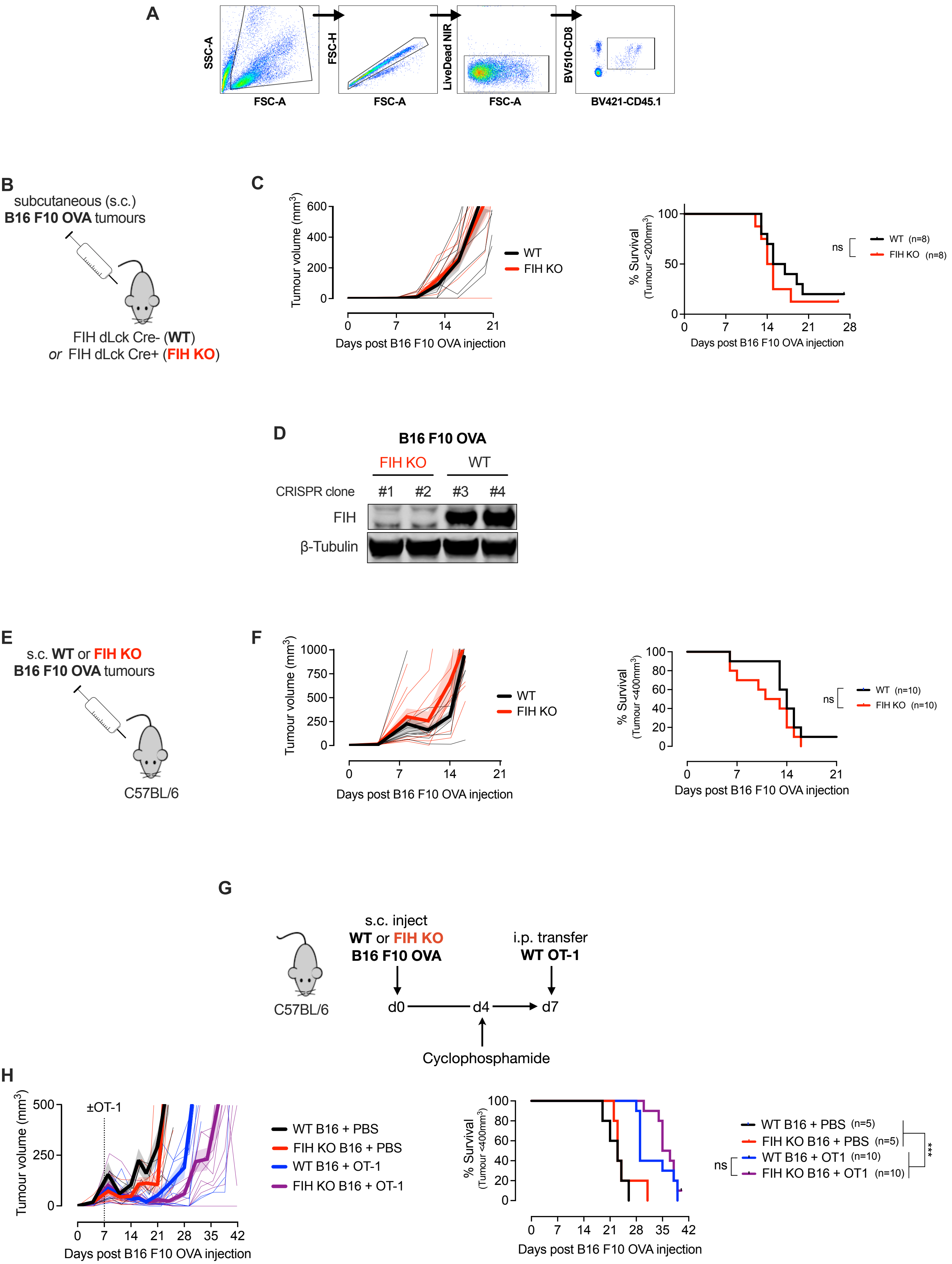
A
SSC-A
FSC-A
FSC-H
FSC-A
LiveDead NIR
FSC-A
BV510-CD8
BV421-CD45.1
B
subcutaneous (s.c.)
B16 F10 OVA tumours
FIH dLck Cre- (WT)
or FIH dLck Cre+ (FIH KO)
C
Tumour volume (mm3)
600
400
200
0
0
7
14
21
Days post B16 F10 OVA injection
WT
FIH KO
% Survival (Tumour <200mm3)
100
50
0
0
7
14
21
28
Days post B16 F10 OVA injection
ns
WT (n=8)
FIH KO (n=8)
D
B16 F10 OVA
FIH KO
WT
CRISPR clone
#1
#2
#3
#4
FIH
β-Tubulin
E
s.c. WT or FIH KO
B16 F10 OVA tumours
C57BL/6
F
Tumour volume (mm3)
1000
750
500
250
0
0
7
14
21
Days post B16 F10 OVA injection
WT
FIH KO
% Survival (Tumour <400mm3)
100
80
60
40
20
0
0
7
14
21
Days post B16 F10 OVA injection
ns
WT (n=10)
FIH KO (n=10)
G
C57BL/6
s.c. inject
WT or FIH KO
B16 F10 OVA
i.p. transfer
WT OT-1
d0
d4
d7
Cyclophosphamide
H
Tumour volume (mm3)
±OT-1
500
250
0
0
7
14
21
28
35
42
Days post B16 F10 OVA injection
WT B16 + PBS
FIH KO B16 + PBS
WT B16 + OT-1
FIH KO B16 + OT-1
% Survival (Tumour <400mm3)
100
80
60
40
20
0
0
7
14
21
28
35
42
Days post B16 F10 OVA injection
WT B16 + PBS (n=5)
FIH KO B16 + PBS (n=5)
WT B16 + OT1 (n=10)
FIH KO B16 + OT1 (n=10)
ns
***

Supplemental Figure 4: *In vivo* outcomes following FIH loss in tumour or T cells.
(A) Flow cytometry plots of gating strategy to identify WT and FIH KO OT-I T cells using CD45.1/2.2 congenic markers. **(B)** *In vivo* orthotopic experiment - *HIF1an*$^{fl/fl}$ *dLck* Cre negative ('WT') and *HIF1an*$^{fl/fl}$ *dLck* Cre positive ('FIH KO') mice were injected subcutaneously with B16 F10 OVA melanoma cells and tumour growth was measured over time. **(C)** Tumour volume and survival (threshold: tumour <200 mm^3) of WT or FIH KO mice with B16 F10 OVA tumours (n=8 mice per group). **(D)** Immunoblot of FIH protein levels in CRISPR-generated B16 F10 OVA FIH wild-type (WT) and knockout (FIH KO) clones. **(E)** *In vivo* orthotopic experiment - C57BL/6 wild-type mice received a subcutaneous injection of FIH wild-type or FIH knockout B16 F10 OVA cells and tumour volume was measured over time. **(F)** Tumour volume and survival (threshold: tumour <400 mm^3) in C57BL/6 wild-type mice were injected subcutaneously with WT or FIH KO B16 F10 OVA cells (n=10 mice per group). **(G)** Timeline of *in vivo* adoptive transfer immunotherapy experiment - C57BL/6 mice received a subcutaneous injection of WT or FIH KO B16 F10 OVA cells, lymphodepleted with cyclophosphamide on day 4 and then received an intraperitoneal injection of PBS or wild-type OT-I T cells on day 7. Tumour growth was monitored over time. **(H)** Tumour volume and survival (tumour<200 mm^3) on mice injected with WT or FIH KO B16 F10 OVA tumour cells after intraperitoneal injection of PBS (n=5) or WT OT-I T cells (n=10 mice). Data are representative of one **(H)** or two **(C,F)** independent experiments. Line represents mean, shaded area represent s.e.m **(C,F,H)**. Log-rank test **(C,F,H)**. ***p<0.01; ns, non-significant.
